# Characteristics of women age 15-24 at risk for excess weight gain during pregnancy

**DOI:** 10.1371/journal.pone.0173790

**Published:** 2017-03-14

**Authors:** Tammy Chang, Michelle H. Moniz, Melissa A. Plegue, Ananda Sen, Matthew M. Davis, Eduardo Villamor, Caroline R. Richardson

**Affiliations:** 1 Department of Family Medicine, Medical School, Institute for Healthcare Policy and Innovation, University of Michigan, Ann Arbor, Michigan, United States of America; 2 Department of Obstetrics and Gynecology, Medical School, Institute for Healthcare Policy and Innovation, University of Michigan, Ann Arbor, Michigan, United States of America; 3 Department of Family Medicine, University of Michigan, Ann Arbor, Michigan, United States of America; 4 Department of Family Medicine and Biostatistics, University of Michigan, Ann Arbor, Michigan, United States of America; 5 Northwestern University Feinberg School of Medicine, Ann & Robert H. Lurie Children’s Hospital of Chicago, Chicago, Illinois, United States of America; 6 Department of Epidemiology, School of Public Health and Center for Human Growth and Development, University of Michigan, Ann Arbor, Michigan, United States of America; University of Tennessee Health Science Center, UNITED STATES

## Abstract

**Purpose:**

Excess weight gain during pregnancy is a serious health concern among young pregnant women in the US. This study aimed to characterize young women at highest risk for gaining over the recommended amount of weight during pregnancy.

**Methods:**

Using a database that is representative of births in large U.S. cities, The Fragile Families and Child Wellbeing Study, we identified mothers of singleton term-infants age 15–24 years at the time of delivery. Institute of Medicine guidelines were used to categorize each mother’s weight gain as less than, within, or more than recommended during pregnancy. Multinomial logistic regression models for weight gain category were performed, controlling for age, race/ethnicity, federal poverty level (FPL), health status, and prepregnancy BMI.

**Results:**

Among the weighted sample (n = 1,034, N = 181,375), the mean (SD) age was 21 (3) years, 32% were black, 39% were Hispanic, 44% reported income under the Federal Poverty Level, 45% were overweight or obese before pregnancy, and 55% gained more weight than recommended during pregnancy. Women who were overweight or obese before pregnancy were at increased risk for gaining more pregnancy weight than recommended, compared to normal-weight women (adjusted Relative Risk Ratio (RRR) = 3.82, p = 0.01; RRR = 3.27, p = 0.03, respectively). Hispanics were less likely than non-Hispanics to gain more weight than recommended (RRR = 0.39, p = 0.03).

**Conclusions:**

The majority of mothers ages 15–24 gained excess weight during pregnancy, a strong risk factor for later obesity. Prepregnancy overweight or obesity and non-Hispanic ethnicity predicted excess pregnancy weight gain. Interventions and policies should target these high-risk young women to prevent excess weight gain.

## Introduction

Weight gain during pregnancy has lasting health implications for both mothers and infants. Excessive weight gain during pregnancy increases the risk of dangerous complications during pregnancy, including diabetes, hypertensive disease, fetal growth abnormalities, operative delivery, and stillbirth, and contributes to the risk of long-term obesity in both mother and child[1–9]. Inadequate weight gain is associated with low birth weight, preterm birth and failure to initiate breastfeeding[10]. As a result, the Centers for Disease Control and Prevention (CDC) and the Institute of Medicine (IOM) have called for more effective interventions to promote healthy weight gain during pregnancy to address this critical public health issue[9,11].

In the US, less than 40% of women gain within the IOM recommended guidelines, while nearly 50% gain over the recommended weight during pregnancy[9,10,12–14]. Promoting healthy pregnancy weight gain among young women is particularly important, as the US has one of the highest rates of adolescent pregnancy among high-income countries and rising rates of obesity among women and adolescents[15–17]. Despite recent declines in teen birth in the US, in 2014, nearly 1 in 15 births in 2014 was to a teenager (age 15–19 years)[18]. As a result, morbidity related to inappropriate weight gain continues to contribute to poor health among at-risk young mothers and their children.

Some interventions designed for adult women have been shown to be effective at maintaining healthy weight gain during pregnancy[19–22]. However, few interventions have been designed specifically for pregnant adolescents or young women. Pregnant adolescents and young women likely have different needs and preferences than pregnant adults, and as a result, interventions for this at-risk group should be tailored to their distinct needs and preferences to be effective. To appropriately tailor interventions, there is a critical need to understand the characteristics of adolescents and young women who are at greatest risk for inappropriate weight gain during pregnancy.

Risk factors for inappropriate pregnancy weight gain are known among adults and prepregnancy BMI, age, and race[9,23,24]. However, the effects of sociodemographic factors on inappropriate weight gain among pregnant adolescents and young women is unclear. The aim of this study is to use a large national sample to identify risk factors for inappropriate weight gain during pregnancy specifically among adolescents and young women.

## Materials and methods

The Fragile Families and Child Wellbeing Study follows a cohort of mother and father dyads and their children born in large US cities between 1998 and 2000 (roughly three-quarters of dyads are unwed parents). “Fragile families” denotes that these families are at greater risk of breaking up and living in poverty than more traditional families[25]. National weights make the data representative of births in the 77 US cities with populations over 200,000[26].

The study extracted information from medical records about mothers’ pregnancy and delivery (including prepregnancy BMI), and the mother’s weight gain during pregnancy. Follow-up interviews gathered additional information, including access to and use of healthcare and childcare services, experiences with local welfare and child support agencies, parental conflict and domestic violence, and child health and well-being. Because this data set provides a unique glimpse into the many factors that may affect weight gain during pregnancy, it has the potential to be used to create a typology of pregnant adolescents and young women who gain appropriate weight versus those who do not.

The cohort of interest was pregnant women between the ages of 15 and 24 at time of delivery to capture adolescents as they transitioned into adulthood[27]. Data was further restricted to those who had medical record information available and carried singleton pregnancies to term (37 weeks or later). Mothers were categorized into BMI categories using prepregnancy height and weight information. Prepregnancy BMI among those who were 18 or older were categorized using standard cut-offs (<18.5 = underweight, 18.5–25 = normal, 25–30 = overweight, 30–40 = obese, 40+ = morbidly obese) and those between the ages of 15–17 were categorized using age-specific percentiles from the CDC (<5^th^ percentile = underweight, 5^th^-85^th^ percentile = normal, 85^th^-95^th^ = overweight, 95^th^+ = obese, BMI of 40+ = morbidly obese)[28,29]. IOM guidelines updated in 2009 for gestational weight gain vary by prepregnancy BMI: underweight women should gain between 28 and 40 lbs., normal weight between 25 and 35 lbs., overweight between 15 and 25 lbs. and obese and morbidly obese between 11 and 20 lbs.[9]. The guidelines have been shown to be appropriate for both adults and adolescents[12]. These guidelines were used to categorize each mother’s weight gain as less than recommended, within recommended, or more than recommended during pregnancy.

Factors that contributed to an increased likelihood of gaining more weight than IOM guidelines recommended were assessed with weight gain category (less than recommended, within recommended, more than recommended) during pregnancy as the outcome. Bivariate analyses, Chi-squared tests and ANOVA as appropriate, were performed evaluating factors potentially associated with pregnancy weight gain including baseline age, socioeconomic status (SES), race, ethnicity, federal poverty level (FPL), health status, insurance status, marital status, situational history variables (homelessness, inadequate money, living with biological father), poor nutrition, alcohol use, tobacco use, other drug use, medical comorbidities, preexisting medical conditions, time when prenatal care began, and prepregnancy BMI category.

The final adjusted multinomial logistic regression model included factors that were significant in the bivariate models or have been shown to be associated with weight gain during pregnancy in the IOM’s conceptual framework[9]. These included age, race, ethnicity, SES, current health and prepregnancy BMI category as covariates. Interactions between covariates, primarily between prepregnancy BMI and age, were also investigated. All analyses were adjusted using national survey weights with STATA version 13.1 (StataCorp LP, College Station, TX). The University of Michigan IRB determined this study to be not regulated.

## Results

There were 1,413 mothers between the ages of 15 and 24 in the national sample with available medical record data. Mothers who carried multiples (n = 20), gave birth prior to 37 weeks (n = 142) or had unknown gestational age at delivery (n = 3) were excluded from analysis. An additional 214 mothers were removed due to missing either prepregnancy BMI or weight gain during pregnancy data, leaving a final unweighted analytic sample size of 1,034.

The average age of the weighted sample was 21 years (SD = 3.0) with roughly equal distribution across races and a large proportion (39.0%) being Hispanic and falling below the FPL (43.9%). Nearly half of mothers had a normal BMI (49.1%), while another quarter (26.3%) were overweight prior to becoming pregnant (Table 1).

**Table 1:** Demographic characteristics of sample population, Fragile Families and Child Wellbeing Study by gestational weight gain category.

	Unweighted, n(%)	Weighted, %	Gestational weight gain			Chi-squared P-value
			% under, (17.7%)	% within, (27.2%)	% Over, (55.2%)	
**Age, n = 1,034**						0.66
15–16	23 (2.2)	3.1	1.9	1.7	4.2	
17–18	163 (15.8)	14.2	12.0	12.2	15.8	
19–21	491 (47.5)	49.8	44.8	54.8	48.9	
22–24	357 (34.5)	32.9	41.3	31.3	31.1	
Mean(SD)	20.6 (2.1)	20.6 (3.0)				
**Race, n = 1,023**						0.95
White	337 (32.9)	36.2	39.7	34.4	35.9	
Black	444 (43.4)	32.3	26.0	34.6	33.1	
Other	242 (23.7)	31.5	34.3	31.0	31.0	
**Ethnicity, n = 1,033**						0.26
Non-Hispanic	666 (64.5)	61.0	50.8	57.1	66.2	
Hispanic	367 (35.5)	39.0	49.2	42.9	33.8	
**Federal Poverty Level, n = 1,034**						0.92
0–49%	233 (22.5)	23.0	21.2	21.1	24.5	
50–99%	192 (18.6)	20.9	29.2	17.9	19.7	
100–199%	287 (27.8)	26.7	23.8	28.0	27.0	
200–299%	176 (17.0)	15.6	17.4	17.7	14.0	
300%+	146 (14.1)	13.8	8.4	15.3	14.8	
**Health Status, n = 1,031**						0.68
Great	310 (30.1)	23.2	24.5	28.5	20.3	
Very Good	388 (37.6)	40.0	38.2	32.3	44.4	
Good	274 (26.6)	26.5	32.8	27.4	24.0	
Fair/Poor	59 (5.7)	10.3	4.5	11.8	11.3	
**Prepregnancy BMI Category, n = 1,034**						0.002
Underweight	63 (6.1)	5.5	10.2	8.2	2.7	
Normal	528 (51.1)	49.1	65.2	61.4	37.9	
Overweight	237 (22.9)	26.3	9.3	16.0	36.8	
Obese	178 (17.2)	16.6	11.8	11.7	20.5	
Morbidly Obese	28 (2.7)	2.5	3.5	2.7	2.1	

Based on IOM guidelines over half (55.2%) of the weighted sample gained more than recommended during pregnancy, 27.2% gained within guidelines, and 17.7% gained less than recommended. Bivariate analysis found prepregnancy BMI to be the only factor significantly associated with weight gain category (p-value = 0.002), with overweight and obese individuals having higher probabilities of gaining more than the recommended weight compared with other categories. (Table 1)

A multinomial logistic regression model including age, race, ethnicity, federal poverty level, current health and prepregnancy BMI category as covariates did not find any significant associations between gaining less than recommended compared to within recommended guidelines (Table 2).

**Table 2:** Multinomial logistic regression model results.

	Over Recommended vs. Within		Under Recommended vs. Within	
Predictor	RRR (95% CI)	p-value	RRR (95% CI)	p-value
**Age**	0.94 (0.81, 1.11)	0.497	1.09 (0.87, 1.37)	0.453
**Race**				
White	Reference			
Black	0.60 (0.22, 1.67)	0.318	0.58 (0.15, 2.25)	0.421
Other	1.70 (0.62, 4.66)	0.292	0.77 (0.22, 2.74)	0.680
**Hispanic**	**0.39 (0.17, 0.90)**	**0.029**	1.23 (0.39, 3.83)	0.716
**Poverty Level**	0.91 (0.67, 1.22)	0.506	0.80 (0.52, 1.24)	0.308
**Health**	1.09 (0.51, 2.32)	0.816	1.09 (0.48, 2.46)	0.834
**BMI Category**				
Underweight	0.41 (0.08, 2.20)	0.289	1.21 (0.18, 7.97)	0.839
Normal	Reference			
Overweight	**3.82 (1.39, 10.49)**	**0.011**	0.53 (0.10, 2.68)	0.427
Obese	**3.27 (1.15, 9.26)**	**0.027**	0.87 (0.24, 3.10)	0.852
Morbidly Obese	1.39 (0.05, 42.67)	0.846	1.43 (0.03, 71.53)	0.852

Prepregnancy BMI category (p-value = 0.04) and ethnicity (p-value = 0.03) were significantly associated with gaining more than recommended compared to within recommended guidelines. Overweight and obese individuals had higher likelihoods of gaining more than recommended, when compared with normal weight individuals (Relative Risk Ratio (RRR) (95% CI) = 3.82 (1.39, 10.49) and 3.27 (1.15, 9.26), respectively). Hispanics were less likely to gain over the recommended weight (RRR (95% CI) = 0.39 (0.17, 0.90)) than non-Hispanics. Interactions between prepregnancy BMI category and other covariates including age, race, ethnicity, self-reported health and federal poverty level were not found to be significant.

**Fig 1:**
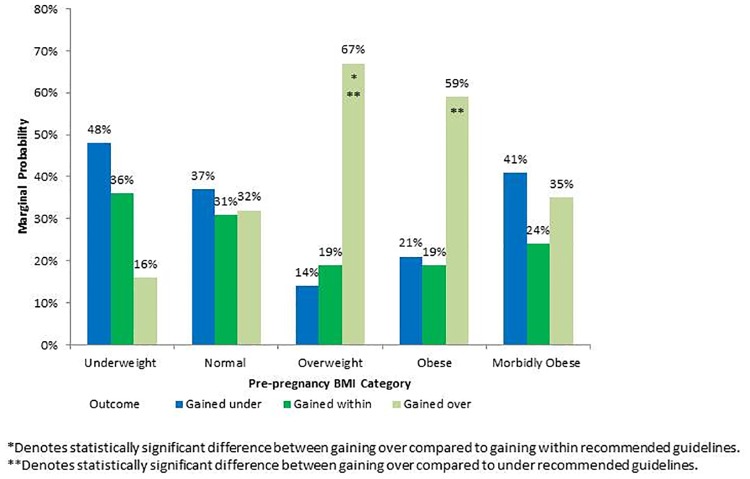
Marginal probabilities of gaining under, within, or over IOM guidelines by prepregnancy BMI.

The marginal probability of each outcome category was estimated across prepregnancy BMI categories using the final model (Fig 1). Mothers who were overweight or obese prior to getting pregnant had substantially higher probabilities of gaining more than the recommended guidelines, compared to normal-weight women. The other prepregnancy BMI groups showed no significant difference between probabilities of gaining under, within or over the recommended guidelines.

## Discussion

Categorical weight gain was similar in our sample of 15–24 year-old women compared to cohorts that include older women (under IOM guidelines: 18% vs 21%; within: 27% vs 32%; over: 55% vs 47%)[30]. Overweight and obese pregnant young women were more likely to gain excess weight during pregnancy compared to normal weight peers, while Hispanic young women were less likely than non-Hispanic peers to gain excess weight during pregnancy. These findings are also consistent with previously reported national and state-level data that included older adults[9,14,31]. No characteristic was associated with gaining too little weight compared to gaining within the guidelines.

Though 15 year olds are arguably different than 24 year olds, younger mothers in our sample did not have a different risk for excess weight gain than older mothers. This finding highlights the importance of prepregnancy BMI at any age for predicting weight gain during pregnancy. Interestingly, Hispanic women in this study were found to be less likely to gain excess weight. Future studies that aim to understand the reason for this distinction could help develop interventions among those at elevated risk for excess weight gain. Our findings also have important implications for the care of pregnant adolescents, young women, and their children today.

First, BMI is objective data that is readily obtained during usual clinical care and can be easily used to identify young pregnant women at-risk for gaining excess weight during pregnancy. There is no need for complicated tests or a prolonged screening process. Based on a Cochrane Systematic Review, high-quality evidence indicates that diet or exercise, or both, during pregnancy can reduce the risk of excessive weight gain in pregnancy[32]. Once at-risk women are identified, available resources to improve diet and exercise habits can be provided to prevent long-term morbidity associated with excess weight gain. Next, pregnancy has been shown to be a time when women are particularly activated and engaged in their health[33,34]. Overweight and obese adolescents and young women would likely benefit from support and resources to achieve healthy diet and exercise habits regardless of pregnancy status. During pregnancy, young women often have increased number of clinical visits and community support which can lead to greater opportunities to address these important issues.

In addition to intrauterine changes that may occur with excess weight gain in pregnancy, poor health habits are often passed on from mothers to their infants[34–36], creating an inter-generational cycle of poor health among these at-risk families. As a result, accurate information, resources and support to achieve healthy weight gain is particularly important for overweight and obese pregnant adolescents and young women. Studies have shown that clinicians may be uncomfortable discussing weight gain with pregnant women[37,38], but that pregnant women are interested in receiving more information about this topic[39]. Adolescent pregnant women in particular may have an even higher need for counseling due to the increased prevalence of body dissatisfaction among this age group[40]. Interventions and programs must be designed that take adolescent-specific factors into account.

Finally, obese adolescents have also been found to be significantly less likely to use birth control compared to normal weight adolescents, despite having similar levels of sexual activity[40]. Efforts to provide responsible education and resources to all adolescents to prevent unintended pregnancy can have greater effects beyond contraception; these efforts may also reduce the risk of long-term morbidity associated with obesity among this at-risk group.

## Limitations

Our findings are subject to certain limitations. First, these data are cross-sectional and cannot be used to infer causation. BMI data was extracted from medical records and may be either patient self-report or anthropometric measurements. These data were also collected in 1998 and 2000 and may no longer represent epidemiologic patterns seen today. However, our study uses a nationally representative sample of births among at-risk adolescents and young women, and includes many social factors that have been hypothesized to be related to weight gain during pregnancy. Finally, our sample may not be sufficiently powered to examine interaction effects as described in our methods, though the results with our current sample (n = 1,034) found only small effects that did not approach significance.

## Conclusion

Prepregnancy BMI and ethnicity are important factors that predict excess pregnancy weight gain among adolescents and young women age 15–24. Interventions and policies should focus on high-risk women to prevent excess weight gain-related morbidity among these mothers and their children.
